# Characterization of clustered bacteriocin-type signal domain protein genes in *Treponema denticola*

**DOI:** 10.3389/fdmed.2025.1543535

**Published:** 2025-02-19

**Authors:** Tomoyuki Nukaga, Eitoyo Kokubu, Kazuko Okamoto-Shibayama, Yuichiro Kikuchi, Masahiro Furusawa, Takashi Muramatsu, Kazuyuki Ishihara

**Affiliations:** ^1^ Department of Endodontics, Tokyo Dental College, Tokyo, Japan; ^2^ Department of Microbiology, Tokyo Dental College, Tokyo, Japan; ^3^ Department of Operative Dentistry, Cariology, and Pulp Biology, Tokyo Dental College, Tokyo, Japan

**Keywords:** *Treponema denticola*, periodontitis, bacteriocin, clustered lipoprotein, bacteriocin ABC transporter

## Abstract

**Background:**

Periodontitis is caused by the dysbiosis of subgingival plaque, and *Treponema denticola* is the pathogen associated with this disease. Bacteriocins are involved in interbacterial competition during dysbiosis. In our previous study, three potential bacteriocin ABC transporter genes (*tepA1-B1, tepA2-B2,* and *tepA3-B3)* of *T. denticola* were investigated. Upstream of *tepA1-B1,* three genes annotated as bacteriocin-type signal domain proteins are located. However, the role of these proteins in *T. denticola* remains unclear. In the present study, these bacteriocin-type signal domain proteins were characterized to elucidate their putative roles in *T. denticola*.

**Methods:**

Gene clusters surrounding bacteriocin-type signal domain protein genes were compared in silico. The expression of proteins and transporters was evaluated using real-time quantitative reverse transcription PCR (qRT-PCR). Bacteriocin-type signal domain proteins were detected using immunoblot analysis. The expression of bacteriocin-like proteins was investigated by co-culturing with *Treponema vincentii*.

**Results:**

The DNA sequences of the bacteriocin-type signal domain protein genes and upstream lipoprotein genes were highly conserved. Expression of the bacteriocin-type signal domain protein and *tepA1* was slightly higher in the mid-log phase than in the stationary phase and was reduced upon co-culture with *T. vincentii*. Bacteriocin ABC transporter gene *tepA1* was expressed independently of *tepA2* and *tepA3*. Immunoblot analysis detected bacteriocin-like proteins in culture supernatants. However, bactericidal activity was not detected in the culture supernatant of *T. denticola*.

**Conclusion:**

Three tandem lipoprotein-bacteriocin-type signal domain protein genes may have originated from duplication. Bacteriocin-type signal domain proteins are expressed under unstimulated conditions and are secreted by *T. denticola* cells.

## 1 Introduction

Periodontal disease is an inflammation of the periodontal tissue caused by subgingival plaque bacteria (1). A major etiologic agent is a microbiome alteration called dysbiosis, which is the shift in the composition of the microbiome to one with a large number of virulent microorganisms. *Treponema denticola* is an anaerobic, spiral-shaped microorganism (2) isolated from chronic periodontitis lesions in high abundance, and is a major disease pathogen (3, 4). A recent study identified *T. denticola* as an index strain for dysbiosis (5).

**Figure 1:**
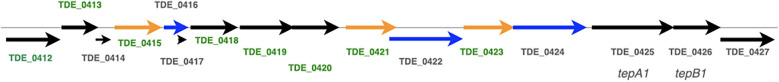
Bacteriocin-type signal domain proteins and flanking region. TDE_0416, TDE_0422, and TDE_0424 contained bacteriocin signal sequences. TDE_0425 and TDE_0426 are potential bacteriocin ABC transporters.

A large number of microbial taxa have been detected in dental plaque (6). Competition and symbiosis between microorganisms play key roles in their survival in dental plaques (7). In multispecies biofilms, bacteria produce toxic components, such as H_2_O_2_ and bacteriocins, to inhibit other microorganisms (8). *T. denticola* must evade the attack from other bacteria and communicate to other bacteria during dysbiosis. Bacteriocins are important weapons to overcome competitors (9) and many gram-positive bacteria, including *Streptococcus mutans* and *Streptococcus salivarius*, produce these molecules (10, 11). Bacteriocin-like effects have been reported for several periodontopathic bacteria (12–14). In a recent metagenomic analysis of the periodontitis microbiome, bacteriocins were reported as the second most abundant biosynthetic gene cluster (15). However, the specific genes involved in the interactions between periodontopathic bacteria remain unclear. Among the genome of periodontopathic bacteria, *T. denticola* has the largest number of annotations of “bacteriocin.” In our previous study, *tepA2*, whose deduced amino acid sequence is similar to that of the bacterial immunity protein (ImmA) of *S. mutans* was detected in the genome sequence of *T. denticola*. *tepA2* is a putative bacteriocin ABC transporter, an ATP-binding/permease protein, and *TepB2*, which is located downstream of *tepA2,* is a putative bacteriocin ABC transporter, a bacteriocin-binding protein (16). These proteins might contain an ABC transporter in the cytoplasmic membrane of *T. denticola*. Additionally, two sets of other genes annotated as bacteriocin ABC transporters (*tepA1-tepB1*, and *tepA3-tepB3*) were detected. Among the three bacteriocin ABC transporters, the *tepA1-tebB1* region has three upstream genes encoding bacteriocin-type signal domain proteins (bacteriocin-like proteins TDE_0416, TDE_0422, and TDE_0424) (Figure 1)*.* These genes contain a bacteriocin-type signaling domain, although their functions remain unknown. Exported proteins may play a role in bacterial interactions.

In the current study, we investigated the function of these cluster genes, focusing on the genes encoding bacteriocin-like proteins upstream of *tepA1* to investigate the fate of proteins and their role in *T. denticola*.

## 2 Materials and methods

### 2.1 Analysis of the genes upstream of *tepA1*

The similarity of the genes flanking the bacteriocin-like protein genes against the genes and proteins in the database of the National Center for Biotechnology Information was determined using blast search (https://blast.ncbi.nlm.nih.gov/Blast.cgi). Multiple sequence alignments were performed using ClustalW (17) and phylogenetic trees were constructed using the neighbor-joining method in GENETYX_MAC v.21 (NIHON SERVER, Tokyo, Japan). For a comparison of protein structures, the AlphaFold Protein Structure Database (https://alphafold.ebi.ac.uk) was used.

### 2.2 Bacterial strain and culture conditions

The strains used in this study are listed in Table 1. These strains were obtained from the American Type Culture Collection or culture collection in our laboratory. *T denticola, Treponema socranskii*, and *Treponema vincentii* were maintained in TYGVS medium (20) containing tryptone (Becton Dickinson, Sparks, MD, USA), yeast extract (Becton Dickinson), gelatin (Becton Dickinson), volatile fatty acids, and rabbit serum (Nihon biotest, Tokyo, Japan) under anaerobic conditions (80% N_2_, 10% H_2_, and 10% CO_2_) in an anaerobic chamber (ANX-3, Hirasawa, Tokyo, Japan). To detect the bacteriocin-type signal domain protein of *T. denticola* in the culture supernatant, *T. denticola* was inoculated into TYGVHS medium, which is a TYGVS medium containing 2% horse serum (Nihon Biotest, Tokyo, Japan) instead of 10% rabbit serum. For *T. denticola* KT-3, 40 µg/mL erythromycin was added to the TYGVS medium. *Aggregatibacter actinomycetemcomitans, Actinomyces,* and *Streptococcus* were maintained on Tryptic soy agar (Becton Dickinson Sparks, MD) containing hemin (5 µg/mL), menadione (0.5 µg/mL), and 10% defibrinated horse blood (Nihon Biotest).

**Table 1:** Strains used in this study.

Strain	Description	Source or reference
*Treponema denticola* ATCC 33520	wild-type	American Type Culture Collection
*Treponema denticola* ATCC 33521	wild-type	American Type Culture Collection
*Treponema denticola* ATCC 33404	wild-type	American Type Culture Collection
*Treponema denticola* ATCC 35405	wild-type	American Type Culture Collection
*Treponema denticola* GM1	wild-type	(16)
*Treponema denticola* KT-3	*tepA2*::Em	(16)
*Treponema socranskii* ATCC 35536	wild-type	American Type Culture Collection
*Treponema vincentii* ATCC 35580	wild-type	American Type Culture Collection
*Actinomyces viscosus* ATCC 15987	wild-type	American Type Culture Collection
*Aggregatibacter actinomycetemcomitans* Y4	wild-type	(18)
*Streptococcus mutans* MT8148R	wild-type	(19)
*Streptococcus sanguinis* ATCC 10556	wild-type	American Type Culture Collection
*Streptococcus oralis* ATCC 10557	wild-type	American Type Culture Collection

### 2.3 Analysis of the expression of bacteriocin-like proteins and bacteriocin transporter-like proteins

To detect the gene encoding the bacteriocin-like protein in *T. denticola* strains, Southern blotting was performed as described previously (16). Genomic DNA of *T. denticola* was isolated using a Gentra Pure Gene Kit (Qiagen, Tokyo, Japan). Genomic DNA was digested with *Hind* III, separated on a 1% agarose gel, and transferred onto a Nytran N blotting membrane (Cytiba, Tokyo, Japan). A digoxygenin-labeled probe (504 bp) was prepared using a Thermal Cycler C1000 (Bio-Rad Laboratories, Hercules, CA, USA) with primer pairs 416_probeF and 416_probeR listed in Table 2.

**Table 2:** Primers and TaqMan probes used in this study.

Primer/probe	Sequence
416_probeF	5′-ATGAAAAAATTTATTAAATTGACGGATG-3′
416_probeR	5′-TTAGTTATACTTTTTTGTATAAGCTTCA-3′
TDE0416F	5′-GCTACCGGTGTACATATGAAACCAT-3′
TDE0416R	5′-GCCTCTTTGCCCTTCTTTTCCTTTA-3′
TDE0416P	5′-CCCATCCGGTTTCCCA-3′
TDE0422F	5′-CGCAATCAAACCTTTAGAGCAAATGAA-3′
TDE0422R	5′-CATGTATTGAAATAGATTGTTTTCCTGTTAAGTCA-3′
TDE0422P	5′-CAAGCCCGTATCTTTC-3′
TDE0424F	5′-CGCAATCAAACCTTTAGAGCAAATGAA-3′
TDE0424R	5′-CATGTATTGAAATAGATTGTTTTCCTGTTAAGTCA -3′
TDE0424P	5′-CAAGCCCGTATCTTTC-3′
*tepA1*F	5′-TGCCGTGCAAATGACTCTCT-3′
*tepA1*R	5′-TTTTAAAACTGCCTACCCAATAAACGC-3′
*tepA1*P	5′-CACAGCTTGGAACTTT-3’
*tepB1*F	5’-TGAAAAAATTATGGCTTGAAGCACTTGA-3’
*tepB1*R	5’-TGCCATATCTGCCTTGTATTTTAACTCT-3’
*tepB1*P	5’-CCTGCAACAGCAATTC-3’
*tepA3*F	5′-CACTCCTGTATTGTTGAAAGTCTTAACG-3′
*tepA3*R	5′-CACTTACGATTTTAAACTCGGCTCTT-3′
*tepA3*P	5′-TTGGGTGCCGAATCTA-3′
*tepB3*F	5′-AGAAAGTTTAAAACTTTTTACAGTCTATGCTCCT-3′
*tepB3*R	5′-CATTATCCCCGCAGTTAAGAGATGA-3′
*tepB3*P	5′-TTCCTGCACTTCTCCC-3′
16SF	5′-GCCGATGATTGACGCTGATATAC-3′
16SR	5′-CGGACTACCAGGGTATCTAATCCT-3′
16SP	5′-CTCCCCGCACCTTC-3′

To determine the expression of bacteriocin-like proteins, the mRNA levels of TDE_0416, TDE_0422, and TDE_0424 were evaluated using real-time quantitative reverse transcription PCR (qRT-PCR). *T. denticola* ATCC 35405 was harvested at the mid-log or stationary phase, and total RNA was extracted using TRIzol reagent (Sigma-Aldrich, St. Louis, MO). cDNA was synthesized using the SuperScript First-Strand Synthesis cDNA kit (Invitrogen, Carlsbad, CA, USA). The expression levels of TDE_0416, TDE_0422, and TDE_0424 were determined by qRT-PCR using the primers and TaqMan probes listed in Table 2. Five microliters of cDNA were added in a 45 µL reaction mixture containing 25 μL of 2× TaqMan Universal PCR Master Mix, 5 μL of forward primer, reverse primer, and the TaqMan probe listed in Table 2. The cycling conditions were as follows: 95°C for 10 min, followed by 40 cycles at 95°C for 15 s and 60°C for 1 min each using the 7500 Real-Time PCR system (Applied Biosystems, Foster, CA). 16S rRNA was used as an internal control. The expression levels of each gene were normalized using the relative standard curve method.

To investigate the relationship between the expression of the three bacteriocin ABC transporters, *T. denticola* ATCC 35405 and a *tepA2*-deficient mutant were harvested at the mid-log or stationary phase, and the expression of *tepA1, B1, A3*, and *B3* was evaluated using qRT-PCR, as described above. The primers and TaqMan probes used for qRT-PCR analysis are listed in Table 2.

### 2.4 Preparation of antibodies against bacteriocin-type signal domain proteins

Antibodies were prepared as described previously (21). To prepare polyclonal antibodies that recognize bacteriocin-like proteins, the peptide GKPDGYEGKEGQRG, corresponding to the 89th–102nd residues of the deduced amino acid sequence of TDE_0424, which is also present in TDE_0416 and TDE_0422, was used. A Cys residue was conjugated to the N-terminus of the sequence and synthesized using F-moc chemistry. The peptide was conjugated to keyhole limpet hemocyanin using maleimidobenzoic acid N-hydroxysuccinimide ester. The conjugate (0.15 mg) was used to immunize the rabbits (six biweekly injections), and blood was collected 12 weeks after the initial immunization. Immunization and bleeding were performed by Eurofins Genomics (Tokyo, Japan). Antibody reactivity was confirmed by ELISA, using the peptide GKPDGYEGKEGQRG as the antigen.

### 2.5 Detection of bacteriocin-like proteins from the culture supernatant

To determine whether bacteriocin-type signal domain proteins were released from the cells, *T. denticola* was cultured in TYGVHS medium under anaerobic conditions for 4 days. The culture supernatant of *T. denticola* was centrifuged at 12,000×*g* for 15 min. The outer sheath fraction of *T. denticola* was isolated as described previously (21). The culture supernatants and outer sheath fractions were separated with 10%–20% sodium dodecyl sulfate-polyacrylamide gel electrophoresis (SDS-PAGE), as described previously (21). The separated proteins were transferred using the Trans-Blot Turbo system (Bio-Rad, Hercules, CA, USA), and bacteriocin-type signal domain proteins were detected using the primary antibody described above (1/1,000) and a peroxidase-conjugated secondary antibody against anti-rabbit IgG (1/1,000, Bio-Rad) with a snap i.d. 2.0 (EMD Millipore Corporation, Billerica, MA). Signals were developed using a TMB Membrane Peroxidase Substrate (KPL, Gaithersburg, MD, USA).

### 2.6 Effects of co-culture with *T. vincentii* on the expression of bacteriocin-type signal domain proteins

*T. denticola* ATCC 35405 and *T. vincentii* ATCC 35580 were cultured in TYGVS medium separately at 37°C under anaerobic conditions for 2 days. When the absorbance at 660 nm reached approximately 0.3–0.6, 20 ml of each culture was added. The mixed and original cultures were incubated for 2 h. After incubation, the expression levels of TDE_0416, TDE_0422, and TDE_0424, and *tepA1* in both mixed and single cultures were assessed as described above.

### 2.7 Screening of bactericidal activity

The bacteriocin activity was screened according to a previous report with minor modifications (22). *T. denticola* ATCC 35405 was precultured in TYGVS medium for 3 days and 10 µl of culture was spotted on TYGVS agar, which contains 0.8% Noble agar (Becton Dickinson, Sparks, MD, USA) and incubated anaerobically for 5 days. *Actinomyces viscosus* ATCC 15987, *Aggregatibacter actinomycetemcomitans* Y4, *S. mutans* MT8148R, *Streptococcus sanguinis* ATCC 10556, and *Streptococcus oralis* ATCC 10557 were precultured in Tryptic soy broth overnight at 37°C under anaerobic conditions. *T. socranskii* ATCC 35536 and *T. vincentii* ATCC 35580 were precultured in TYGVS broth at 37°C under anaerobic conditions for 3 days. After incubation, cell density was adjusted to OD660 = 0.2, mixed with molten TYGVS agar at 45°C, and overlayed on TYGVS agar, on which *T. denticola* was spotted. The plate was incubated at 37°C anaerobically for an additional 48 h and the presence of an inhibition zone by *T. denticola* was evaluated.

### 2.8 Statistical analysis

Gene expression was compared using the Student's *t*-test. For the effect of co-culture and expression of bacteriocin ABC transporter-like proteins, normality and homogeneity of variance were tested before applying the Student's *t*-test. Statistical significance was set at *p* < 0.05.

## 3 Results

### 3.1 Characterization of the gene cluster around the bacteriocin-type signal domain protein genes

**Figure 2:**
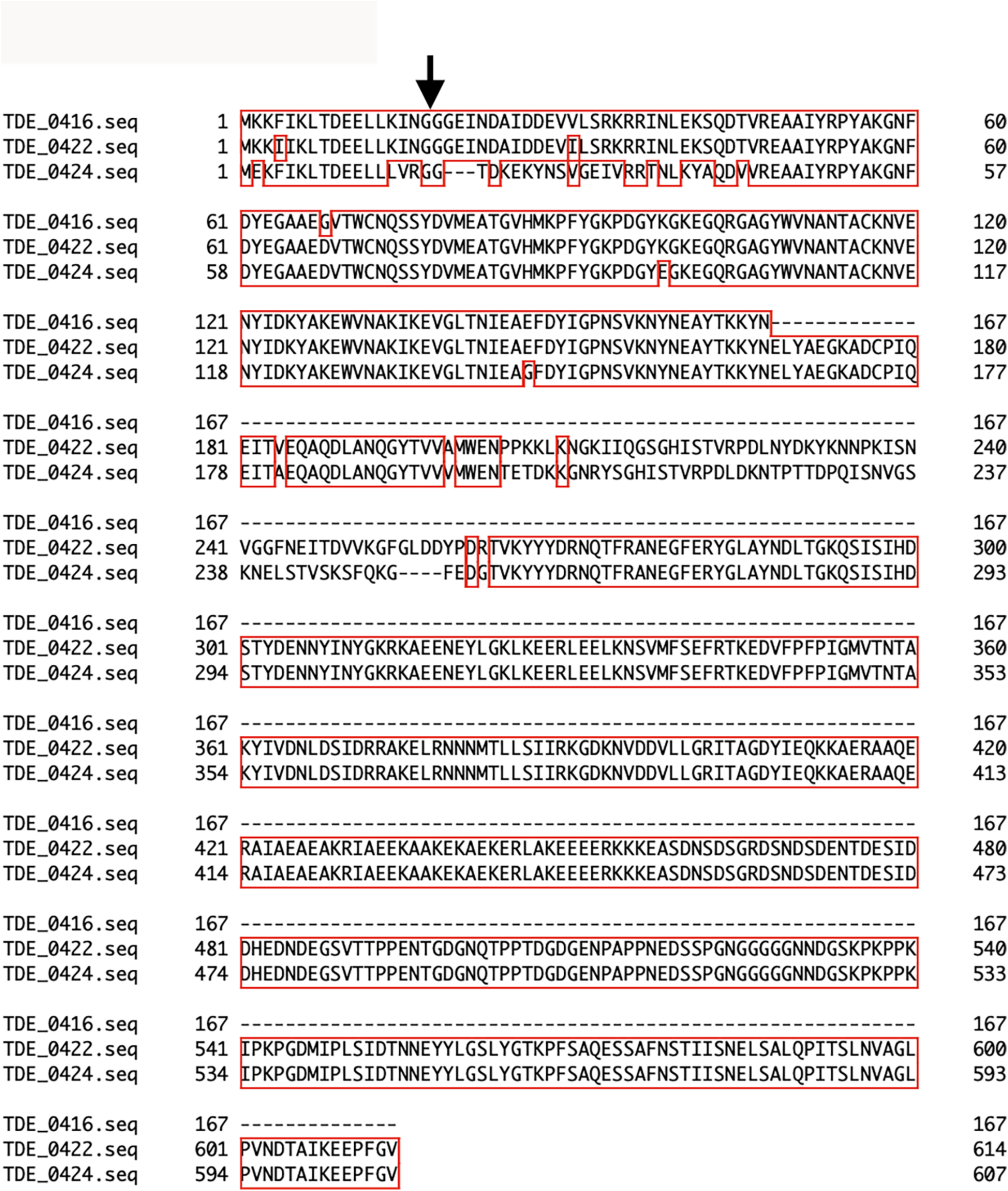
Multiple amino acid sequence alignments of bacteriocin-type signal domain proteins. The arrow indicates a double glycine, which is typical of bacteriocin signal peptides. Alignment of TDE_0416, TDE_0422, and TDE_0424 was performed using Genetyx-Mac 21.2.0.

Three open reading frames encoding the bacteriocin-type signal domain proteins TDE_0416, TDE_0422, and TDE_0424 were located upstream of *tepA1* (TDE_0425, Figure 1). At the DNA level, TDE_416 shared 98% and 96% identity with 501 and 367 bases in TDE_0422 and TDE_0424, respectively. TDE_0422 shared 94% identity with TDE_0424 in 1,714 bases. The deduced amino acid sequences of all open reading frames contained bacteriocin-like signal peptides with double glycine at the 17–18th residues (Figure 2). TDE_0422 and TDE_0424 contain 614 and 607 residues, respectively, whereas TDE_0416 contains 167. TDE_0416 has 98% identity in 167 amino acid residues and 84% identity in 167 amino acid residues with TDE_0422 and TDE_0424, respectively. TDE_0422 shares 89% identity in 616 amino acid residues with TDE_0424. The 459th–522nd amino acid residues of TDE_0422 and the 452nd–515th residues of TDE_0424 were similar to the fibronectin-binding autotransporter adhesin ShdA (PRK15319).

**Figure 3:**
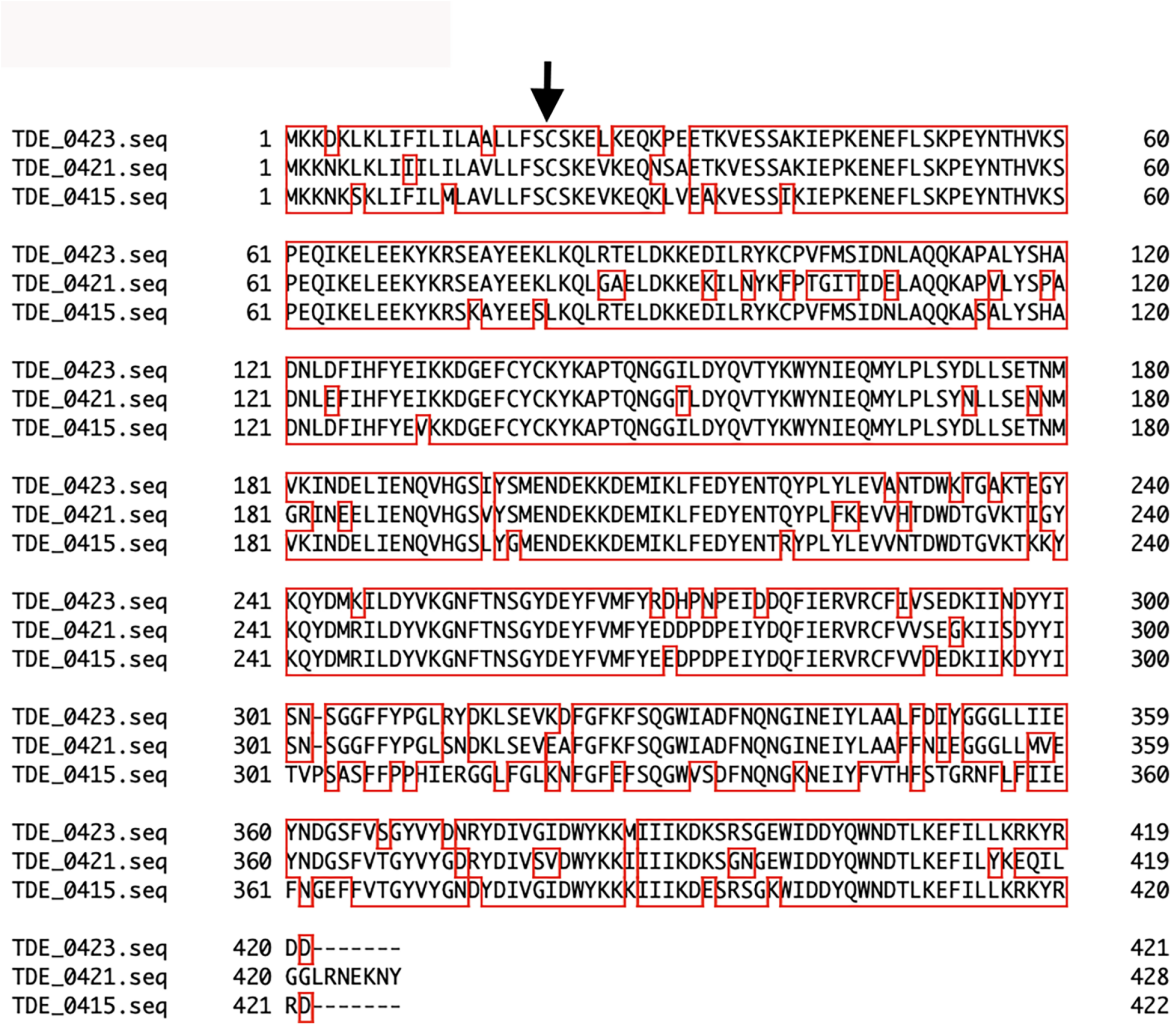
Amino acid sequence alignment analysis of clustered lipoproteins. The arrow indicates the cleavage site of the signal peptidase for lipoproteins as determined using the SignalP program. Alignment of TDE_0415, TDE_0421, and TDE_0423 was performed using Genetyx-Mac 21.2.0.

**Figure 4:**
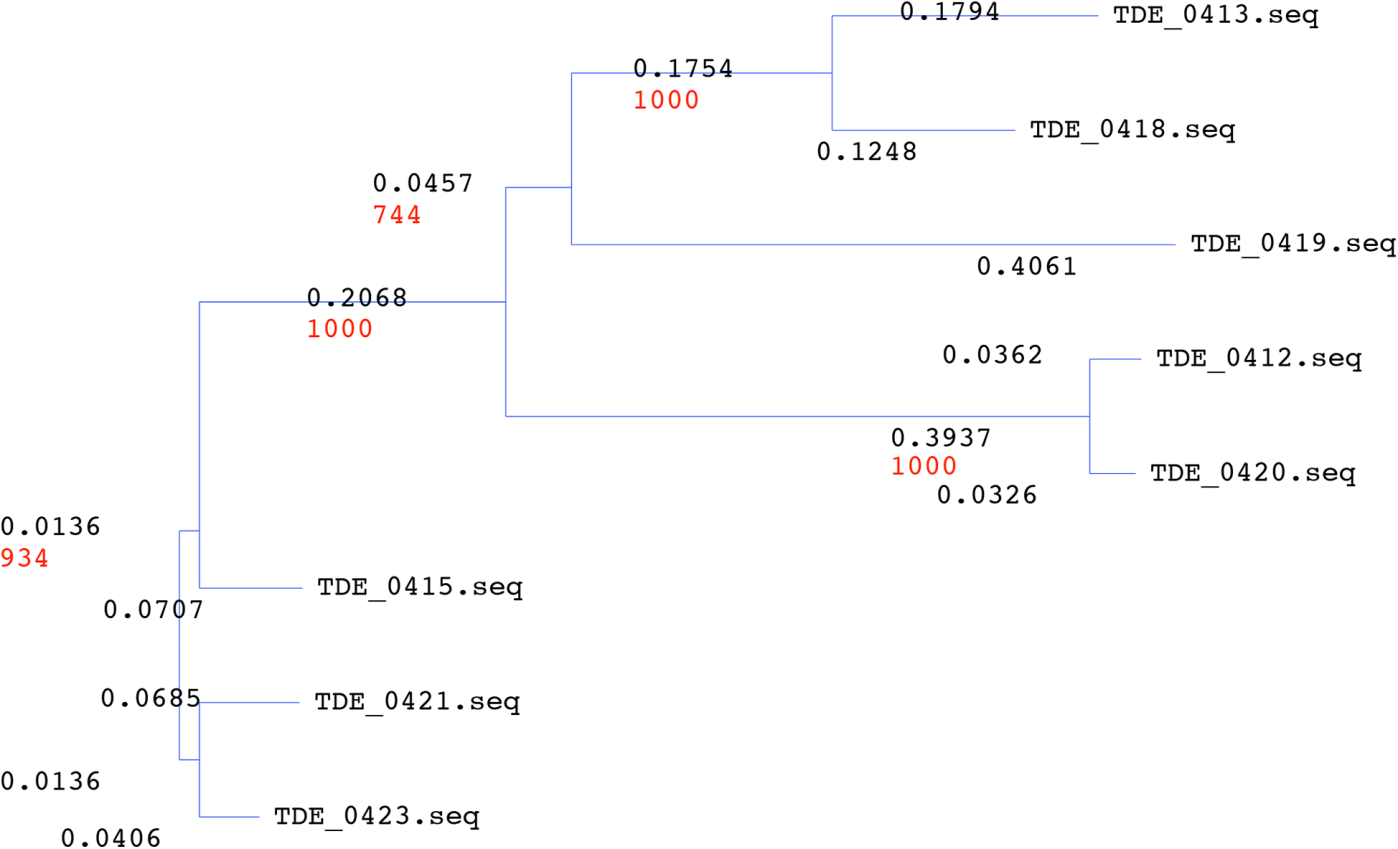
Evolutionary tree of *Treponema denticola* clustered with lipoproteins. The evolutionary tree was organized using the NJ method with 1,000 boost trap trials.

Upstream of *tepA1,* there were eight open reading frames: TDE_0412, TDE_0413, TDE_0415, TDE_0418, TDE_0419, TDE_0420, TDE_0421, and TDE_0423, annotated as *Treponema*-clustered lipoproteins (Figure 1). Known signal sequences of lipoproteins were identified from these sequences, except TDE_0412, using SignalP program (https://services.healthtech.dtu.dk/service.php?SignalP-6.0). TDE_0413 and TDE_0418 were similar to the signal peptide regions of lipoproteins. However, no extensive homology was observed. Other genes were predicted to encode lipoproteins. The amino acid sequence of TDE_0415 showed 78% identity in 404 residues and 83% identity in 422 residues with TDE_0421 and TDE_0423, respectively (Figure 3). The similarity between TDE_0415 and other clustered proteins was less than 30%. The evolutionary tree of the DNA sequences indicated that TDE_0415, TDE_0421, and TDE_0423, located immediately upstream of the three bacteriocin-type signal domain proteins, were closely related (Figure 4). At the DNA level, TDE_0415 shared 85% identity in 1,278 bases and 89% identity in 1,270 bases with TDE_0421 and TDE_0423, respectively. These results suggest that the tandemly arranged genes encoding lipoprotein and bacteriocin-like proteins, TDE_0415-TDE_0416, TDE_0421-TDE_0422, and TDE_0423-TDE_0424, were derived from a common ancestor gene.

**Figure 5:**
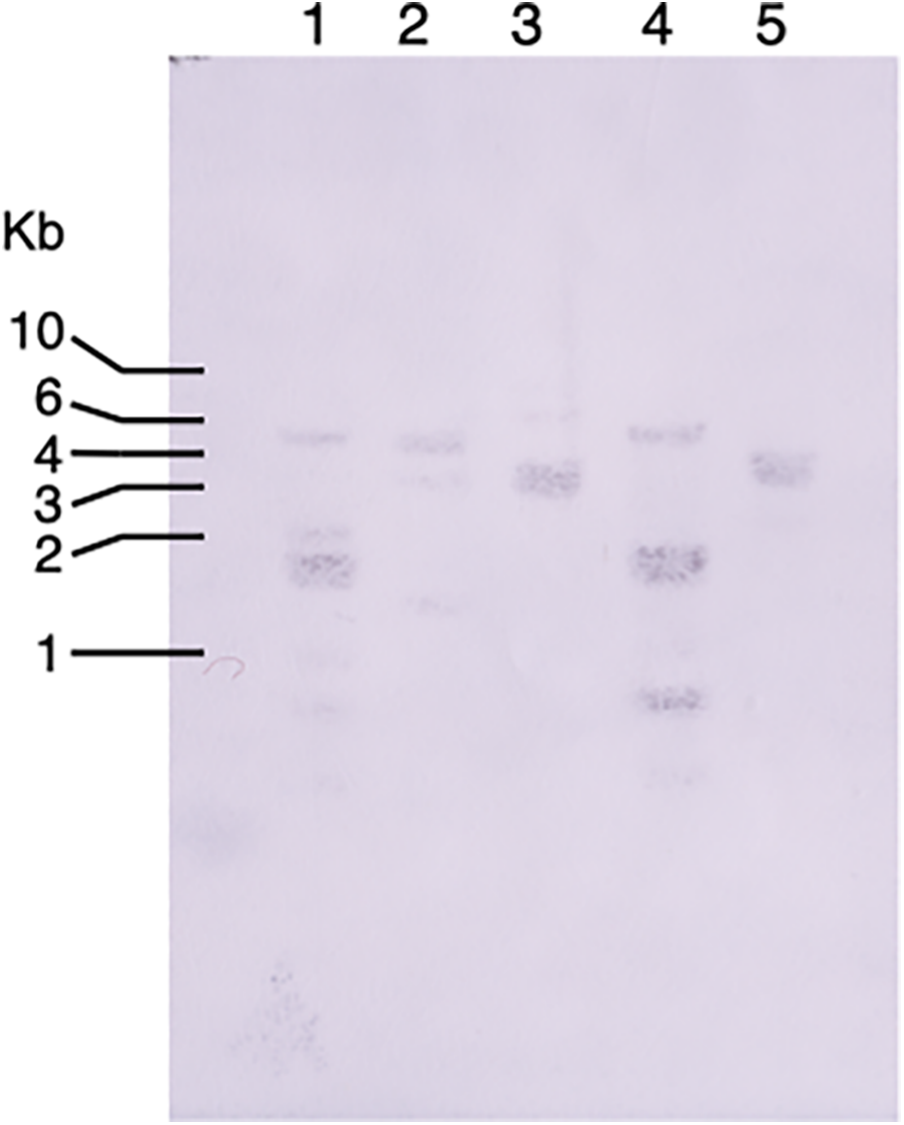
Southern blotting of bacteriocin-type signal domain proteins in *T. denticola* strains. The genomic DNA of *T. denticola* was digested with *Hind* III and the TDE_0416 fragment (504 bp) was used as a probe. Lanes: 1: *T. denticola* ATCC 33520; 2: *T. denticola* ATCC 33521; 3: *T. denticola* ATCC 35405; 4: *T. denticola* ATCC35405; 5: *T. denticola* GM1.

To investigate the prevalence of bacteriocin-type signal domain proteins among *T. denticola* strains, Southern blotting was performed using TDE_0416 as a probe. The results showed that bacteriocin-like proteins are common among *T. denticola* strains (Figure 5). In the AlphaFold Protein Structure Database, the outer membrane efflux protein of *Magnetococcus marinus* and two fimbrial proteins of Bacteroidetes bacteria were detected as structure similarity cluster by Foldseek search using TDE_0424.

### 3.2 Expression of bacteriocin-type signal domain proteins in *T. denticola*

**Figure 6:**
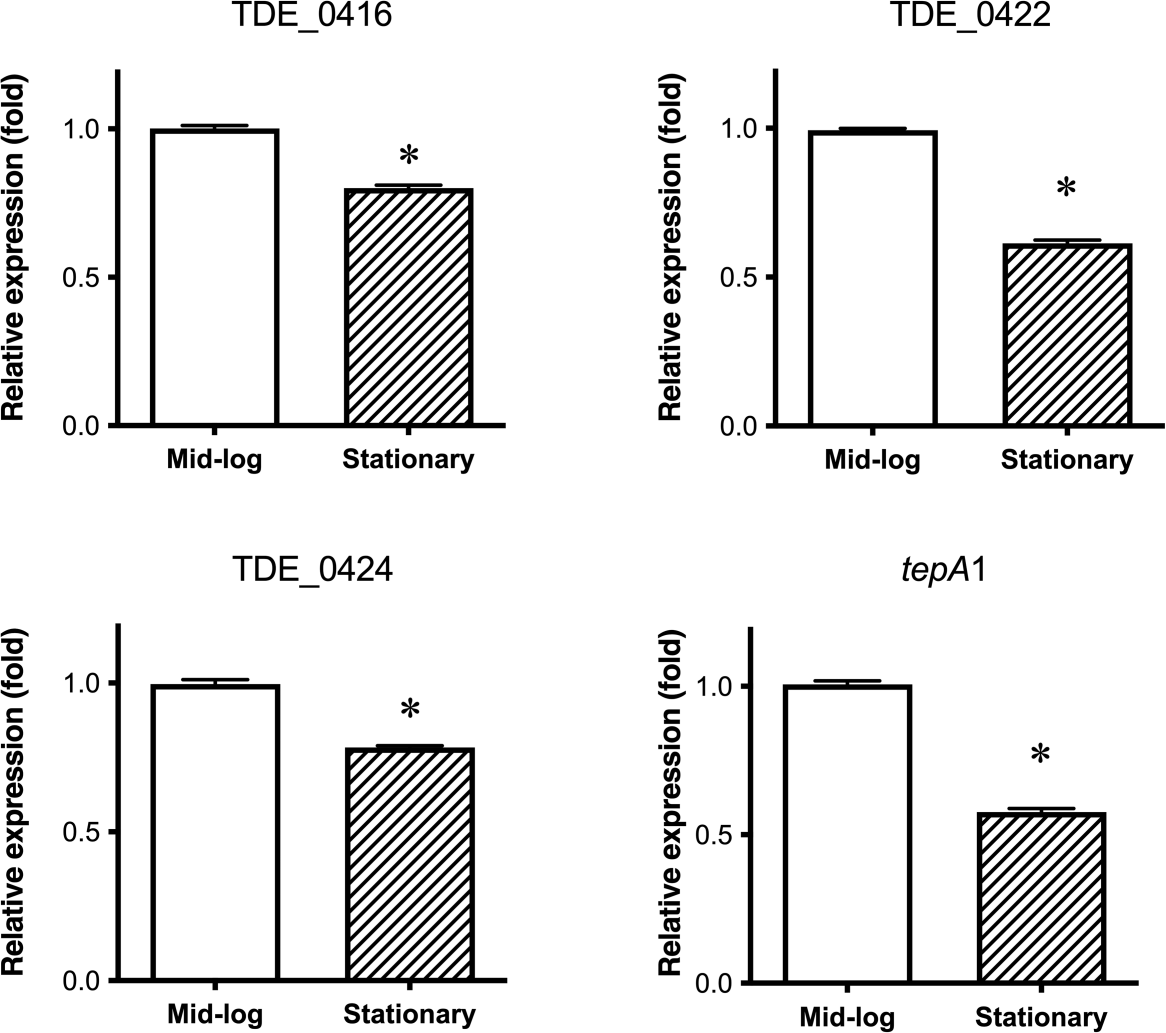
Expression of bacteriocin-type signal domain proteins and transporters in different growth phases. Mid-log: expression in mid-log phase; Stationary: expression in stationary phase. Each dataset is shown as the average fold of mid-log phase ± SD. Representative data (*n* = 3) from three experiments are provided (*P < 0.05).

*T. denticola* was harvested in the mid-log and stationary phases to determine the expression of bacteriocin-type signal domain protein genes. The relative expression of bacteriocin-like proteins to 16S rRNA [mean ± standard deviation (SD)] of TDE_0416, TDE_0422, TDE_0424, and *tepA1* were 1.27 ± 0.014, 0.67 ± 0.003, 0.71 ± 0.013, and 0.11 ± 0.001, respectively. The expression of all genes was slightly decreased in the stationary phase compared to the mid-log phase, but the difference was too small to have a biological effect (Figure 6).

### 3.3 Effects of mixed culture on the expression of bacteriocin-type signal domain proteins

**Figure 7:**
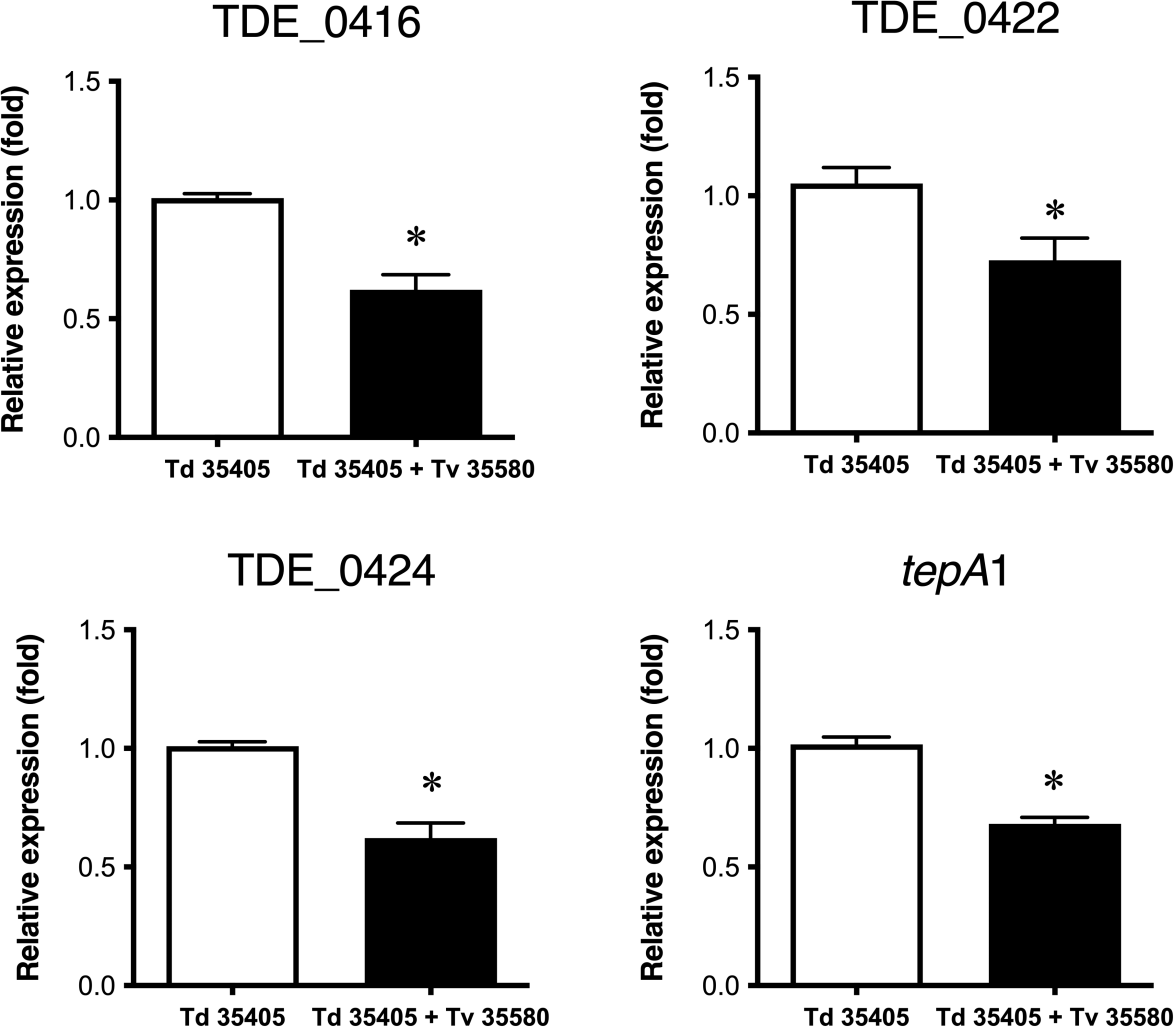
Effect of co-culture on the expression of bacteriocin-type signal domain proteins. Td 35405: single culture of *T. denticola* ATCC 35405; Td 35405 + Tv 35580: co-culture of *T. denticola* ATCC 35405 and *T. vincentii* ATCC 35580. Each dataset is shown as the fold value of the *T. denticola* mono culture ± SD (*n* = 8, *P < 0.05).

Co-culturing *T. denticola* ATCC 35405 with *T. vincentii* ATCC 35580 highlighted the effect of competitive strains on the expression of bacteriocin-like proteins. The expression of TDE_0416, TDE_0422, TDE_0424, and TDE_0425 in *T. denticola* decreased after co-culture with *T. vincentii* (Figure 7). Expression of these genes was not detected in a single culture of *T. vincentii*.

### 3.4 Detection of the bacteriocin-type signal domain proteins in the culture supernatant

**Figure 8:**
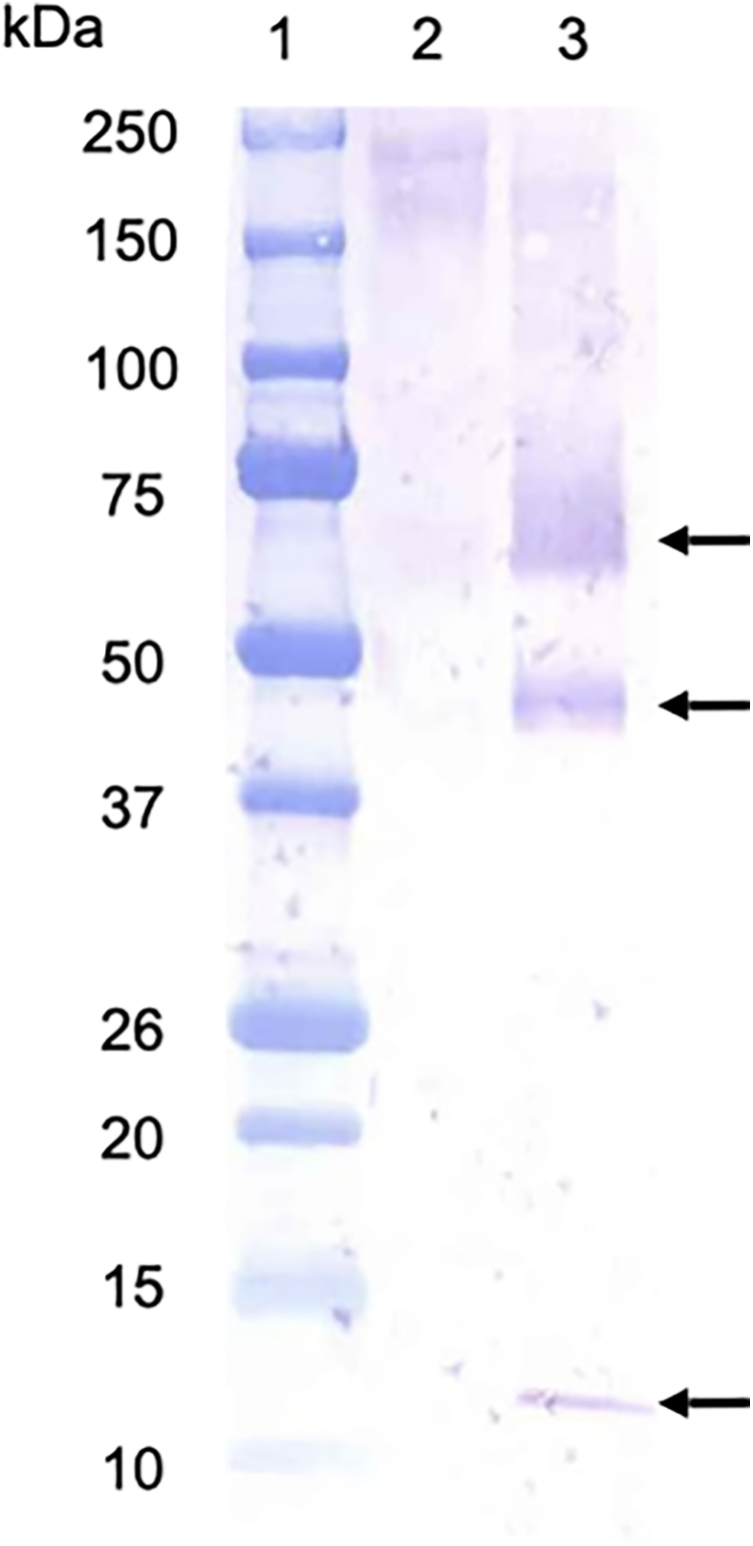
Immunoblot analysis of the culture supernatant of *T. denticola.* Culture supernatant; lane 1, molecular marker; lane 2, TYGVHS medium; lane 3: culture supernatants of *T. denticola* in the same medium. Arrows indicate bands detected using antiserum against bacteriocin-like protein.

To confirm the secretion of bacteriocin-type signal domain proteins from *T. denticola*, an immunoblot analysis was performed using the culture supernatant and outer sheath of *T. denticola* ATCC 35405 and an antibody against the bacteriocin-type signal domain proteins (Figure 8). Bands of approximately 61, 43, and 14 kDa were detected in the culture supernatants*.* The 61 kDa band had a molecular mass close to that calculated from the amino acid sequences of TDE_0422 and TDE_0424 (66,549.77 and 65,688.48, respectively), and the 14 kDa was close to that of TDE_0416 (16,932.39). The 43 kDa protein may be a degraded 61 kDa protein. No bands were detected in the TYGVHS medium*.* No band was detected in the outer sheath fraction (data not shown), indicating that the bacteriocin-type signal domain proteins were not released into the vesicles. These results indicated that bacteriocin-type signal domain proteins were secreted by *T. denticola* cells.

### 3.5 Expression pattern of bacteriocin transporter-like proteins

**Figure 9:**
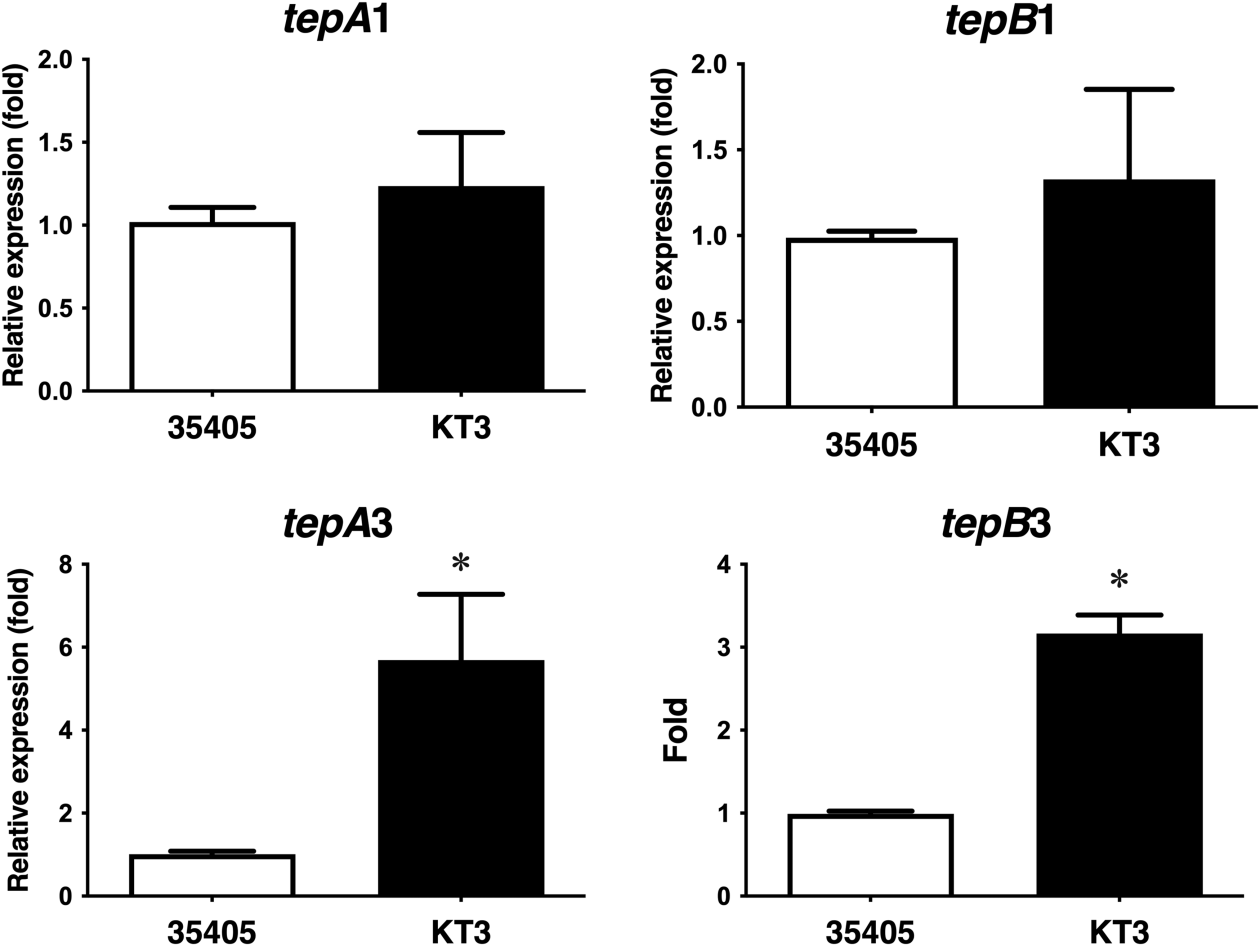
Expression of bacteriocin ABC transporter-like proteins in *T. denticola* ATCC 35405 and *T. denticola* KT-3 (*tepA2* mutant). Expression of *tepA1, B1, A3, and B3* is shown as the average fold of wild-type ± SD (*n* = 9, *P* < 0.05).

In the genome sequence of *T. denticola* ATCC 35405 (https://www.ncbi.nlm.nih.gov/nuccore/AE017226.1/), three bacteriocin-type signal domain proteins (TDE_0416, TDE_0422, and TDE_0424) are located in the same region, whereas only *tepA1* and *tepB1* are located downstream of the bacteriocin-like protein genes. The other two bacteriocin ABC transporter genes (*tepA1-B1* and *tepA3-B3*) are located in a different area without a bacteriocin-like protein gene (16). To investigate the involvement of bacteriocin ABC transporter genes in the export of bacteriocin-like proteins, we evaluated the expression of *tepA1, tepB1, tepA3,* and *tepB3* in a *T. denticola tepA2* gene-inactivated mutant (16) using qRT-PCR*.* As shown in Figure 9, the expression of *tepA3* and *B3* increased in the *tepA2* mutant, whereas the expression of *tepA1* and *B1* was unaffected.

### 3.6 Effects of bacteriocin-type signal domain proteins on the growth of oral microorganisms

To clarify whether bacteriocin-type signal domain proteins possess bactericidal activity, the effects of the culture supernatant on the growth of oral bacteria were evaluated. No growth inhibition was observed for *A. viscosus* ATCC 15987, *A. actinomycetemcomitans* Y4, *S. mutans* MT8148R, *S. sanguinis* ATCC 10556, *S. oralis* ATCC 10557, *T. socranskii* ATCC 35536, or *T. vincentii* ATCC 35580 (data not shown). To investigate the function of the bacteriocin-like protein, we tried to replaced TDE_0423-TDE_0424 with *ermB* via homologous recombination; however, a TDE_0423-TDE_0424 mutant was not obtained.

## 4 Discussion

*T. denticola* has three genes that encode bacteriocin-like proteins (16). These genes and their upstream lipoproteins exhibit high DNA sequence identity. The proteins were constitutively expressed, with a slight decrease in expression during the stationary phase (Figure 6). Although these proteins were secreted into the culture medium, no bactericidal effects were observed.

These three sets of lipoprotein-bacteriocin-like proteins exhibited high DNA sequence identity. Given the high similarity of the three sequences and their locations, it is likely that these gene sets resulted from the duplication of ancestral genes, a phenomenon commonly observed in oral bacteria. In *Porphyromonas gingivalis,* the catalytic domains of arg-gingipain and Hgp44, the C-terminal hemagglutinin/adhesion, are duplicated (23, 24) and Mfa5, a component of the Mfa1 fimbriae, is also duplicated (25). Tandemly arranged genes such as *gtfB* and *gtfC,* are thought to originate via tandem duplication (26, 27). Gene duplications can influence the oligomeric state of the protein (28). Gene duplications are a significant contributor to functional diversity (29). Additionally, bacteriocin-like proteins were detected in all the tested *T. denticola* strains. These findings suggest that these proteins play a physiological role in *T. denticola* and that the three lipoproteins upstream of the protein may be involved in their maturation or function.

The bacteriocin-like signal sequences of TDE_0416, TDE_0422, and TDE_0424, and downstream bacteriocin ABC transporter genes strongly suggested their secretion. Immunoblotting results showed that the sizes of the largest and smallest bands detected in the culture supernatant of *T. denticola* were consistent with those of the bacteriocin-like protein (Figure 8). The vesicles released from many bacteria, including *T. denticola,* export bacterial proteins (30, 31)*.* The vesicles in *T. denticola* contain an outer sheath component of *T. denticola* such as the major outer sheath protein (31). The antibody against bacteriocin-like protein did not react with any proteins in the outer sheath fraction. These results suggest that bacteriocin-like proteins are secreted by bacteriocin ABC transporters. Three bacteriocin ABC transporter-like genes were detected in *T. denticola* ATCC 35405. Conversely, no bacteriocin-type signal domain protein was detected around *tepA2-B2* and *tepA3-B3*. To investigate the relationship among these three ABC transporters, the expression of these genes was evaluated in the *tepA2* mutant. Expression of *tepA3* increased in the *tepA2* mutant, whereas *tepA1* expression remained unaffected (Figure 9). These results suggest that transporters organized by *tepA2* and *tepB2* cooperate with those organized by *tepA3* and *tepB3* and that these four proteins are not involved in the secretion of the bacteriocin-like protein.

Bacteriocin-like proteins showed similarity to the C-terminal domain of ShedA, which forms a beta-barrel structure in the outer membrane (32). The proteins were suggested to be bacteriocins, as their signal peptide resembles that of bacteriocins, and the region showing similarity to the beta-barrel region of ShedA may affect the structure of the outer or cytoplasmic membranes. To investigate the activity of these proteins, we attempted to inactivate TDE_0423 and TDE_0424; however, no mutants were obtained. The DNA sequence of 5′ of TDE_0423 showed high similarity with that of 3′ of TDE_0424. This may have interfered with homologous recombination. Because bacteriocin activity is typically restricted to the gram status of the producer, *T. vincentii* and *A. actinomycetemcomitans* were included as indicator strains for the evaluation of bacteriocin activity. In addition, gram-positive bacteria (*A. viscosus*, *S. mutans*, *S. sanguinis*, and *S. oralis*) were added as indicator strains. The culture supernatant of *T. denticola* ATCC 35405 showed no bactericidal activity against any of the oral bacteria tested. One explanation for this is the culture conditions used. The degree of bactericidal activity against sensitive bacteria is sometimes influenced by specific pH values (33) or by the presence of chemical agents that weaken cell wall integrity (34). The culture conditions may not have been optimal for producing bacteriocin-like proteins*.* Additionally, the spectrum of bactericidal activity may not have included the tested strains.

Another possibility is that these proteins have functions beyond bactericidal effects. Their amino acid sequences were not similar to any bacteriocin except for a double glycine, which is typical of the signal peptide of bacteriocins (35). A previous study identified double glycine-containing signal peptides as precursors of competence-stimulating peptides in *Streptococcus pneumoniae* (36)*.* In *S. mutans,* ﬁrst gene of *ciaRH* operonen coding a double-glycine (GG)-containing peptide acts as a calcium sensing signalling peptide (37). The mRNAs of bacteriocin-like proteins were expressed in *T. denticola,* and their levels slightly decreased in the mid-log phase compared to those in the log phase*.* Protein expression was reduced by co-culturing with *T. vincentii* (Figure 7), indicating that protein expression was regulated by interactions with other microorganisms*.* These proteins were secreted from *T. denticola* cell, and Foldseek analysis showed that structure similarity cluster to the bacteriocin-like protein was membrane protein and a fimbrial protein. It is possible that these proteins are membrane-associated or bind to receptor proteins. Dirix et al. reported that many possible GG motif-containing peptides obtained from a search of gram-negative bacterial genomes showed structural similarities to bacteriocins and peptide hormones (35). These proteins may be peptide hormones. Currently, no information concerning the similarity between the bacteriocin-like proteins and other functional proteins is available. Further analyses of gene expression under various physiological conditions and after gene inactivation are required to clarify the roles of these proteins.

In conclusion, our results show that bacteriocin-like proteins were expressed under unstimulated conditions and secreted from *T. denticola* cells. The expression of these proteins was reduced by co-culturing with *T. vincentii,* suggesting that physiological conditions, such as interactions with other bacteria, regulate their expression*.* The results of the present study shed light on the mechanism of dysbiosis by revealing novel interactions between *T. denticola* and other periodontopathic bacteria and contribute to the understanding of the etiology of periodontitis.

## Data Availability

The raw data supporting the conclusions of this article will be made available by the authors, without undue reservation.
